# Species diversity of environmentally-transmitted bacteria colonizing *Riptortus pedestris* (Hemiptera: Alydidae) and symbiotic effects of the most dominant bacteria

**DOI:** 10.1038/s41598-023-42419-0

**Published:** 2023-09-13

**Authors:** Do-Hun Gook, Minhyung Jung, Soowan Kim, Doo-Hyung Lee

**Affiliations:** Department of Life Sciences, Gachon University, Seongnam-daero 1342, Seongnam-si, Gyeonggi-do South Korea

**Keywords:** Ecology, Microbial ecology

## Abstract

*Riptortus pedestris* (Hemiptera: Alydidae) establish endosymbiosis with specific bacteria from extremely diverse microbiota in soil. To better understand ecology and evolution of the symbiosis, it is important to characterize bacterial species diversity colonizing *R. pedestris* and evaluate their symbiotic effects. Nonetheless, previous research was limited to a few bacteria strains such as *Caballeronia insecticola*. In this study, second-instar nymphs were provided with field soils and reared to adult. Then, bacteria colonizing the midgut M4 region of *R. pedestris* were analyzed for bacterial species identification based on the 16S rRNA gene. First, a total of 15 bacterial species were detected belonging to Burkholderiaceae. Most of *R. pedestris* were found to harbor single bacterial species, whereas several insects harbored at most two bacterial species simultaneously. Among the total insects harboring single bacterial species, 91.2% harbored genus *Caballeronia*. The most dominant species was *C. jiangsuensis*, not previously documented for symbiotic associations with *R. pedestris*. Second, in laboratory conditions, *C. jiangsuensis* significantly enhanced the development, body size, and reproductive potentials of *R. pedestris*, compared to individuals with no symbiotic bacteria. These results add novel information to better understand symbiotic bacteria community establishing in *R. pedestris* and symbiotic effects on the host insects.

## Introduction

The bean bug, *Riptortus pedestris* (Hemiptera: Alydidae), is widely distributed in Asia, and considered as a serious soybean pest, especially in Korea, Japan, and China^1^. This insect can establish endosymbiosis with a group of free-living bacteria in soil especially belonging to family Burkholderiaceae, which colonize the midgut crypts of *R. pedestris*^2^. In the previous studies, at least 19 bacterial strains were reported from *R. pedestris* in Japan^3–6^. Among them, four bacterial strains belonging to the genus *Caballeronia* were used to evaluate their symbiotic effects on *R. pedestris*, and significant symbiotic effects were demonstrated with these bacteria^3,7,8^. Among the bacterial complex, *Caballeronia insecticola* (formerly known as *Burkholderia insecticola*) has received intense attention and is well known for its mutualistic associations with *R. pedestris*. In particular, the gut symbiont has been demonstrated to provide a suite of fitness-related benefits to *R. pedestris*, as well as immunity homeostasis and pesticide resistance^3,9–12^.

*Riptortus pedestris* acquires the symbiotic bacteria from soil, referred to as environmental acquisition or determination^13^, during its early nymphal stages such as second instar^4,14^. This implies that *R. pedestris* would be exposed to extremely diverse microbiota in soil environments that could contain ca. 10^10^ bacteria cells in one-gram soil^15^. Indeed, a laboratory inoculation experiment indicates that *R. pedestris* can establish symbiosis with a wide range of bacterial species in Burkholderiaceae including the model organism, *C. insecticola*^7^. In addition, field surveys demonstrated that soil contained multiple bacterial clades belonging to Burkholderiaceae in South Korea^16^, and seven bacterial genera were detected from the midguts of wild *R. pedestris* individuals^17^. However, the species diversity of symbiotic bacteria, which *R. pedestris* can acquire from soil environment, has not been documented yet.

Understanding the species diversity of symbiotic bacteria in *R. pedestris* can serve as baseline information to elucidate evolutionary and ecological associations between the two groups. The environmental acquisition of symbiotic bacteria by *R. pedestris* is more subject to environmental changes over the course of co-evolution, compared to vertical transmission of the symbionts. In general, co-cladogenesis between insects and their symbionts is entailed by vertical transmission and this relationship becomes robust, whereas evolutionary associations based on environmental acquisition are more reflective and responsive to changes by heterogeneous environments, such as climate, habitat, and geographical differences^13,18,19^. For example, a laboratory experiment showed that *Burkholderia cepacia* complex (BCC) clade in Burkholderiaceae did not stably colonize in a *R. pedestris* population collected in Japan; however, field studies demonstrated that the BCC clade was prevalent in *R. pedestris* populations collected from multiple locations in South Korea^7,16,20^.

Once the species diversity of symbiotic bacteria is revealed, it is important to further evaluate potential symbiotic effects of the colonizing bacteria on *R. pedestris* to understand the ecological relationships between the two groups. The successful establishment of bacteria in the midgut of *R. pedestris* is predicted to accompany fitness changes and subsequently determine adaptive values of the insect in a given environment. As a model system, *C. insecticola* has been demonstrated in a series of studies to substantially enhance multiple facets of *R. pedestris* fitness, compared to insects with no symbiosis^5,8^. However, only limited information is currently available on the symbiotic effects of other bacterial species.

Therefore, in this study, bacterial species diversity colonizing the midgut of *R. pedestris* was characterized using 16S rRNA gene sequencing analysis by providing *R. pedestris* with field soil collected in South Korea. Based on the results of the bacterial identification, the phylogeny of the symbiotic bacteria was addressed. Finally, the most dominant symbiotic species was evaluated for its symbiotic effects on *R. pedestris* with regard to development, growth, and reproduction. This information would serve as baseline information to understand the symbiotic associations of *R. pedestris* with the bacterial complex in nature.

## Results

### PCR analysis for soil samples

From 20 soil samples provided to *R. pedestris*, the two bacterial genera, *Caballeronia* and *Paraburkholderia* were detected from all samples. Genus *Burkholderia* was detected from all samples collected from Gwangju; however, this genus was not detected from 6 out of 10 samples collected from Goesan.

### Gene sequencing information

Among 60 *R. pedestris* reared on field-collected soil, bacterial colonies were successfully isolated from 55 individuals, yielding a total of 165 colonies (Tables 1, 2; Supplementary Table S1). No bacterial colony was formed from five individuals. The isolated colonies were individually subject to sequencing analysis for 16S rRNA gene, and the analysis revealed that all the analyzed colonies belonged to family Burkholderiaceae. The length of bacterial 16S rRNA gene was 1397 ± 3 bp (mean ± SE), with only two cases with less than 1300. The similarity of 16S rRNA gene sequences of the cultured bacteria ranged from 96.2 to 100% with the most relevant type strains in Burkholderiaceae. From the analysis, 10 individuals yielded colonies with the similarity levels equal to or below 98.65%; four individuals yielded colonies from which multiple type strains showed the same, highest similarity within the colony (Supplementary Table S1). These 14 individuals were not included in the following data analysis for bacterial species diversity. Additionally, the pairwise sequence identity analysis classified the 165 bacterial colonies into 24 groups addressing their phylogenetic relationships (Supplementary Fig. S1). The estimated pairwise sequence identities are presented for pairs between individual colonies (Supplementary Table S2) and between phylogenic groups (Supplementary Table S3).

**Table 1:** Symbiotic bacterial species isolated from the midgut of *Riptortus pedestris* when single species was detected across all three cultured colonies.

Insect ID^a^	Accession number	Type bacterial strain matched	Similarity with colony (%)		
			A	B	C
GJ1F1	OQ152651 − 152653	*Caballeronia jiangsuensis*	99.58	99.58	99.58
GJ1F2	OQ152654 − 152656	*Caballeronia jiangsuensis*	99.37	99.58	99.44
GJ2F2	OQ152663 − 152665	*Caballeronia jiangsuensis*	99.58	99.58	99.58
GJ3M3	OQ152675 − 152677	*Caballeronia jiangsuensis*	99.43	99.57	99.57
GJ5F1	OQ152687 − 152689	*Caballeronia jiangsuensis*	99.44	99.51	99.37
GS1F2	OQ152738 − 152740	*Caballeronia jiangsuensis*	99.43	99.50	99.64
GS2M1	OQ152750 − 152752	*Caballeronia jiangsuensis*	99.28	99.36	99.36
GS3F1	OQ152753 − 152755	*Caballeronia jiangsuensis*	99.21	99.21	99.21
GS3M1	OQ152759 − 152761	*Caballeronia jiangsuensis*	99.36	99.36	99.36
GS5F2	OQ152774 − 152776	*Caballeronia jiangsuensis*	99.30	99.41	99.23
GS6F1	OQ152780 − 152782	*Caballeronia jiangsuensis*	99.51	99.51	99.51
GS7F1	OQ152786 − 152788	*Caballeronia jiangsuensis*	99.57	99.43	99.36
GS9F1	OQ152804 − 152806	*Caballeronia jiangsuensis*	99.49	99.28	99.36
GJ1M1	OQ152657 − 152659	*Caballeronia megalochromosomata*	99.78	99.64	99.64
GJ4F1	OQ152678 − 152680	*Caballeronia megalochromosomata*	99.72	99.64	99.64
GJ4M1	OQ152684 − 152686	*Caballeronia megalochromosomata*	99.43	99.43	99.50
GJ6F1	OQ152696 − 152698	*Caballeronia megalochromosomata*	99.57	99.57	99.42
GJ9F1	OQ152720 − 152722	*Caballeronia megalochromosomata*	99.58	99.65	99.58
GS1M1	OQ152741 − 152743	*Caballeronia megalochromosomata*	99.71	99.71	99.71
GS2F1	OQ152744 − 152746	*Caballeronia megalochromosomata*	99.78	99.57	99.57
GS7F2	OQ152789 − 152791	*Caballeronia megalochromosomata*	99.65	99.37	99.37
GS10M1	OQ152732 − 152734	*Caballeronia megalochromosomata*	99.86	99.63	99.71
GJ3M1	OQ152669 − 152671	*C. insecticola* or *C. peredens*^b^	99.37	99.44	99.79
GJ3M2	OQ152672 − 152674	*C. insecticola* or* C. peredens*	99.37	99.79	99.44
GJ5F2	OQ152690 − 152692	*Caballeronia mineralivorans*	99.64	99.64	99.64
GJ7F2	OQ152708 − 152710	*Caballeronia mineralivorans*	99.21	99.15	99.22
GJ6M2	OQ152702 − 152704	*Caballeronia ptereochthonis*	99.28	99.28	99.07
GS1F1	OQ152735 − 152737	*Caballeronia udeis*	99.43	99.64	99.64
GS5F1	OQ152771 − 152773	*Caballeronia fortuita*	99.35	99.08	99.14
GJ2M1	OQ152666 − 152668	*Caballeronia catudaia*	98.91	98.77	98.77
GJ7M1	OQ152711 − 152713	*Caballeronia calidae*	99.00	98.79	98.93
GJ9F2	OQ152723 − 152725	*Paraburkholderia kirstenboschensis*	99.70	99.55	99.70
GJ7F1	OQ152705 − 152707	*Burkholderia ambifaria*	99.72	99.79	99.93
GJ9M1	OQ152726 − 152728	*Pandoraea iniqua*	100	99.86	99.86

^a^*GJ* Gwangju, *GS* Goesan, *F* female, *M* male. ^b^*C. insecticola* and *C. peredens* are listed together because the16S rRNA sequence is known identical for the two species.

**Table 2:** Symbiotic bacterial species isolated from the midgut of *Riptortus pedestris* when two species were detected from the three cultured colonies.

Insect ID^a^	Accession number	Type bacterial strain matched	Similarity with colony (%)		
			A	B	C
GJ5M1	OQ152693 − 152695	*Caballeronia megalochromosomata*	–	–	99.64
		*Paraburkholderia metrosideri*	99.00	99.00	–
GJ6M1	OQ152699 − 152701	*Caballeronia ptereochthonis*	99.07	99.01	–
		*Caballeronia fortuita*	–	–	99.21
GJ10F1	OQ152642 − 152644	*Caballeronia udeis*	99.58	–	–
		*Caballeronia cordobensis*	–	99.37	99.44
GS3F2	OQ152756 − 152758	*Paraburkholderia metrosideri*	98.70	–	–
		*Paraburkholderia agricolaris*	–	98.71	98.71
GS4F1	OQ152762 − 152764	*Caballeronia ptereochthonis*	–	98.93	98.79
		*Caballeronia fortuita*	99.07	–	–
GS6M1	OQ152783 − 152785	*Caballeronia jiangsuensis*	99.36	99.50	–
		*Paraburkholderia metrosideri*	–	–	99.00
GS8F2	OQ152798 − 152800	*Paraburkholderia metrosideri*	–	98.70	98.70
		*Paraburkholderia agricolaris*	98.72	–	–

^a^*GJ* Gwangju, *GS* Goesan, *F* female, *M* male.

### Bacterial species diversity in *R. pedestris* midgut

A greater number of bacterial species were found from *R. pedestris* provided with soil collected from Gwangju, compared to Goesan. A total of 14 bacterial species were found from Gwangju, whereas seven species were detected from Goesan (Supplementary Fig. S2). In both regions, *C. jiangsuensis* and *C. megalochromosomata* were dominant. Between sexes, 11 and eight bacterial species were detected from females and males, respectively. The two genera, *C. jiangsuensis* and *C. megalochromosomata*, were also dominant for both sexes (Supplementary Fig. S2). As presented below, the data were pooled across regions and sexes to estimate bacterial species diversity in *R. pedestris* based on a larger sample size.

**Figure 1:**
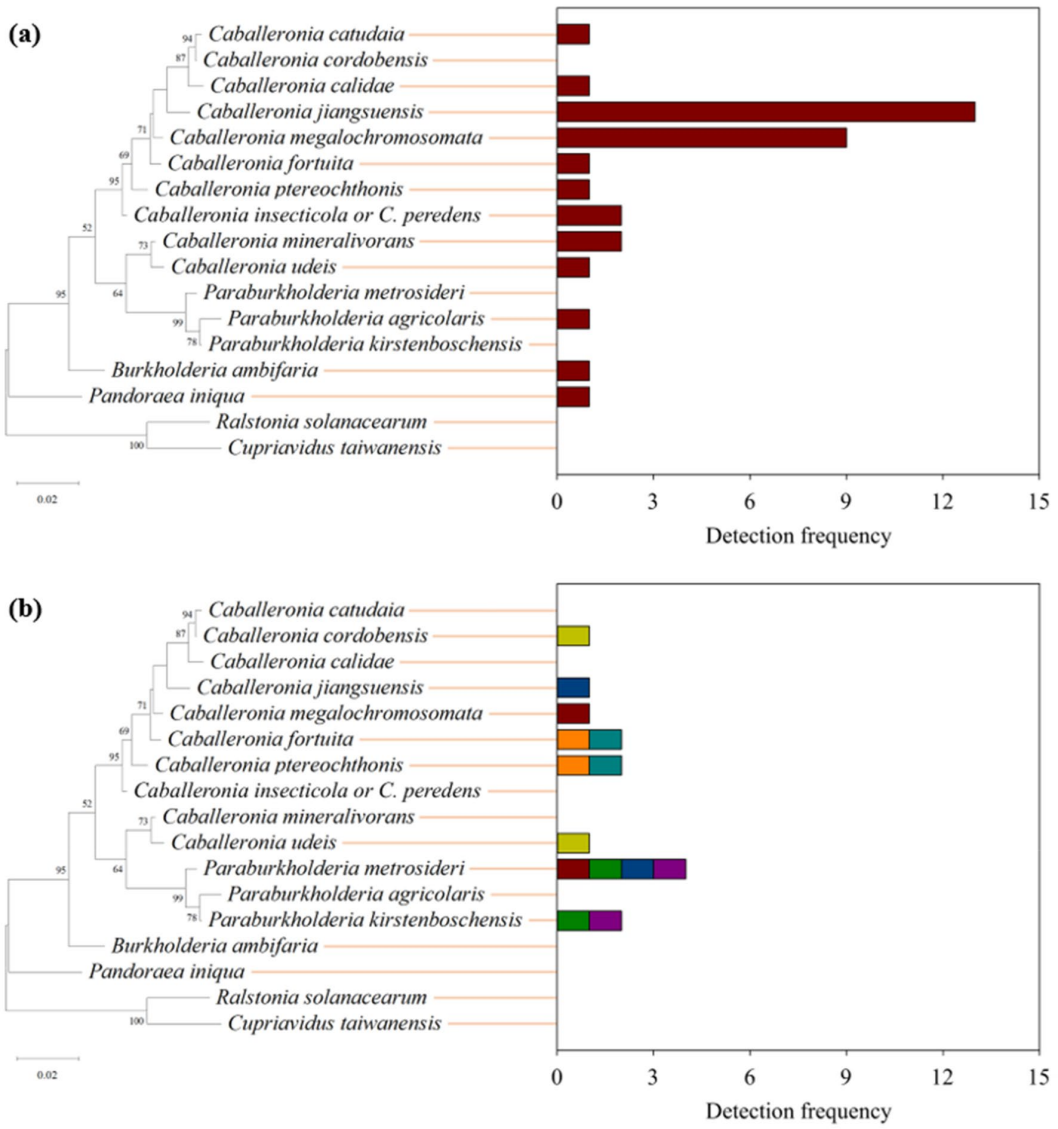
Species diversity of bacterial species isolated from the midgut of adult *Riptortus pedestris* harboring one (**a**) and two (**b**) species of the bacteria. Phylogenetic tree was generated based on 16S rRNA sequences of the matched type strains, and the bootstrap values > 40% are depicted at the nodes. *Ralstonia solanacearum* and *Cuproavidus taiwanensis* were used as outgroup taxa. Note that detection frequency indicates the number of times being detected from insects and the same colors in the panel b indicate two bacterial species isolated from the same individual insect. *Caballeronia insecticola* and *C. peredens* are listed together because their 16S rRNA sequences are known identical.

Single bacterial species was most commonly detected from the midgut of *R. pedestris*, consisting of 34 out of 41 individuals included in the species diversity analysis (Table 1; Fig. 1a). Seven individuals were found to harbor two bacterial species simultaneously in their midguts (Table 2; Fig. 1b). There was no case in which three bacterial species were found from an individual. A total of 15 bacterial species were identified from the midguts of 41 *R. pedestris* analyzed. Among *R. pedestris* detected with single bacterial species, 91.2% harbored bacteria belonging to genus *Caballeronia* in their midguts (Fig. 1a). The most dominant species was *C. jiangsuensis* (38.2%), followed by *C. megalochromosomata* (26.5%), *C. insecticola* or *C. peredens* (5.9%), and *C. mineralivorans* (5.9%). From *R. pedestris* harboring two bacterial species, genus *Caballeronia* was also commonly detected; however, no obvious dominance was observed at species level among the bacteria (Fig. 1b).

### Symbiotic effects on *R. pedestris*

**Figure 2:**
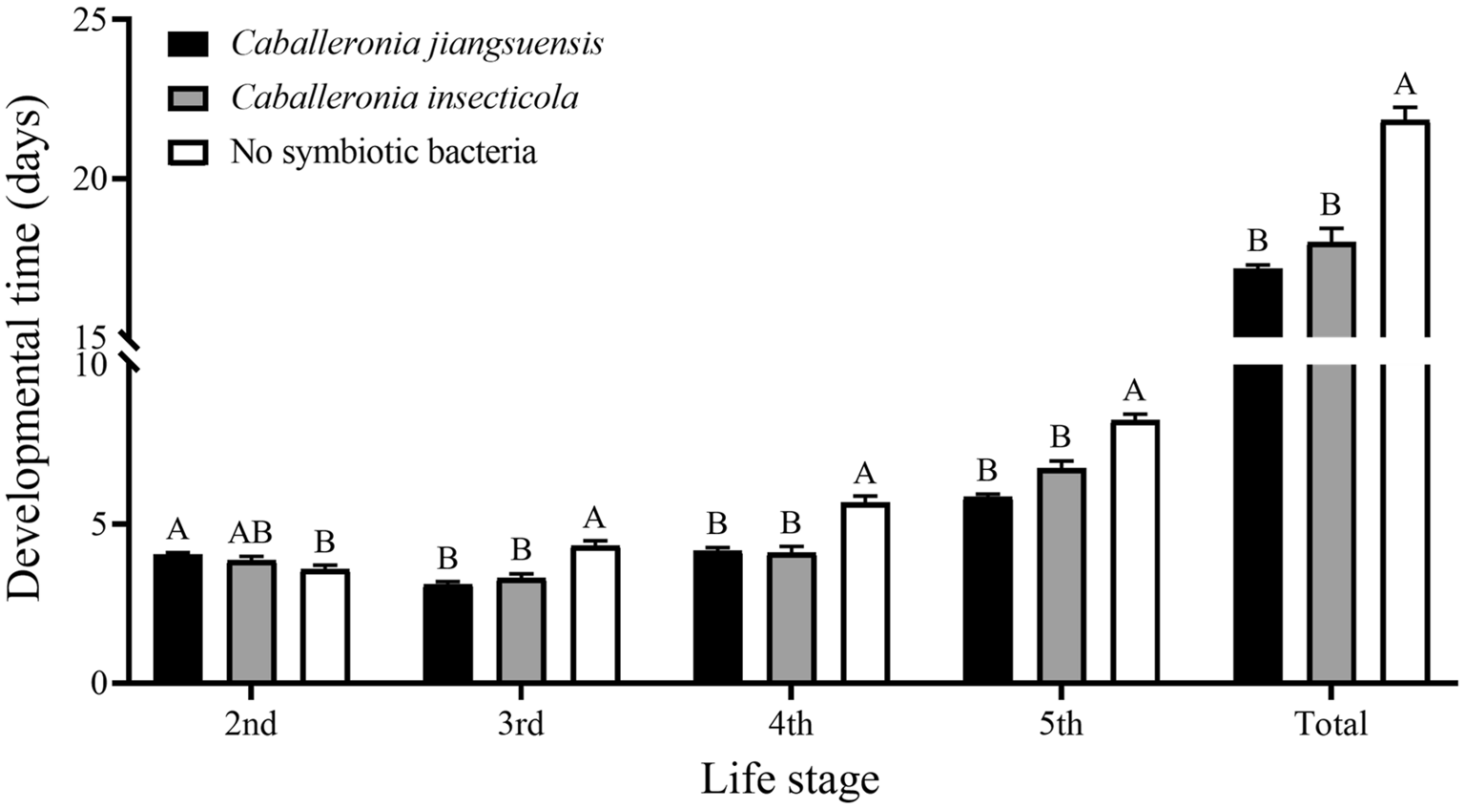
Development time (days) (mean ± SE) of *Riptortus pedestris* inoculated with *Caballeronia jiangsuensis*, *C. insecticola*, and no symbiotic bacteria. Different letters indicate significant difference among the treatments within each nymphal stage (*P* < 0.05). Note that symbiotic bacteria were provided during the second-instar period.

Based on the results above, *C. jiangsuensis* was selected as the most dominant bacterial species established in *R. pedestris*. In general, the nymphal development time was significantly shorter for *R. pedestris* inoculated with *C. jiangsuensis* GJ1F1a [OQ152651], compared to apo-symbiotic individuals (ANOVA; *P* < 0.0001; Fig. 2). However, this was not the case for the second-instar stage, during which *R. pedestris* acquired the bacteria provided (F = 4.87; *df* = 2, 55; *P* < 0.05; Fig. 2). Similar to *C. jiangsuensis*, nymphs inoculated with *C. insecticola* also exhibited significantly faster development, compared to apo-symbiotic individuals (ANOVA; *P* < 0.0001; Fig. 2). There was no significant difference in the development time between the two groups inoculated with *C. jiangsuensis* and *C. insecticola*.

**Figure 3:**
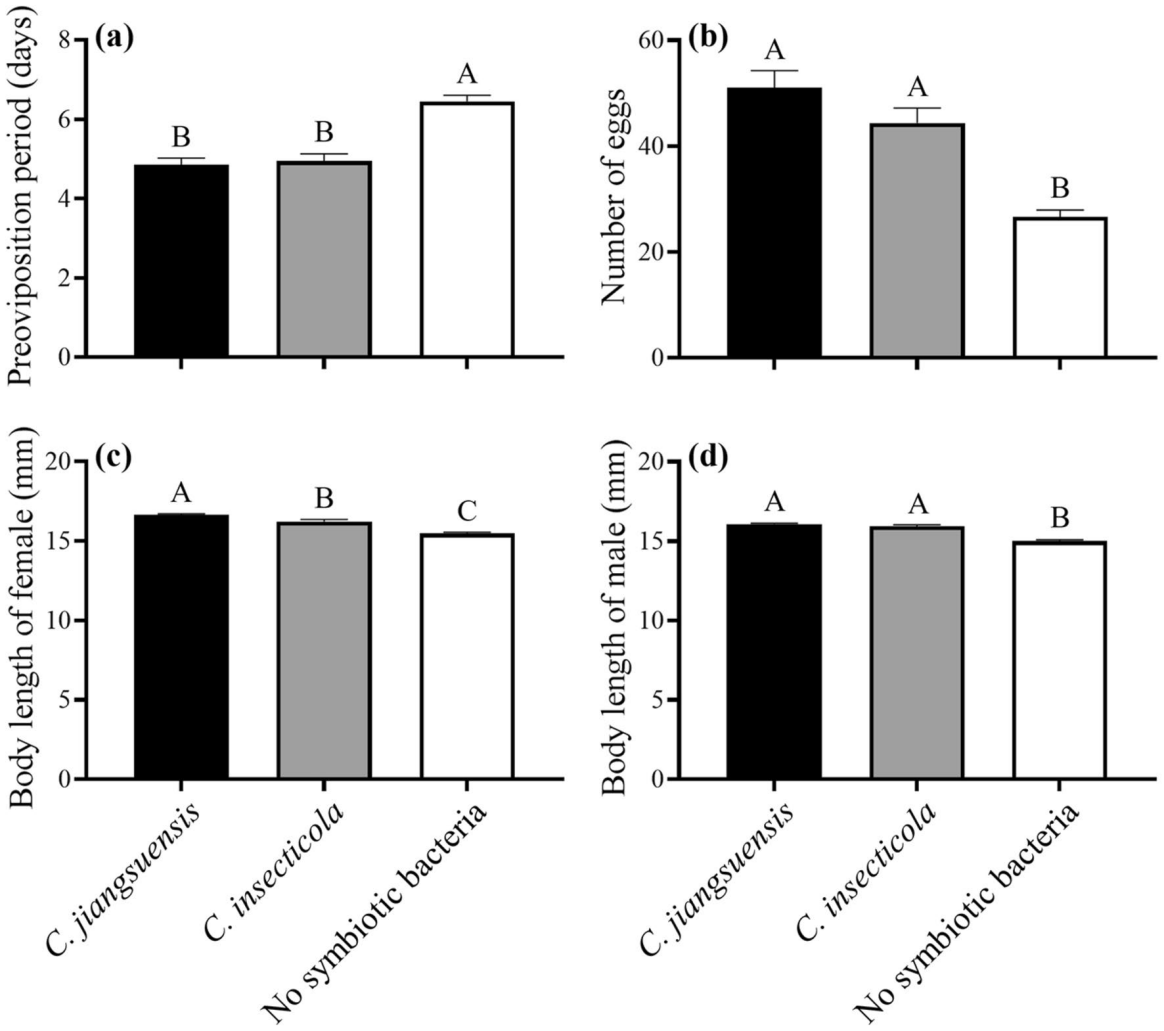
Reproductive potentials (mean ± SE) of female *Riptortus pedestris* (**a**,**b**) and body lengths (mean ± SE) of female (**c**) and male (**d**) inoculated with *Caballeronia jiangsuensis*, *C. insecticola*, and no symbiotic bacteria. Different letters indicate significant difference among the treatments (*P* < 0.05).

The inoculation with *C. jiangsuensis* significantly increased the reproductive potentials of female *R. pedestris*, compared to apo-symbiotic group, yielding shorter preoviposition period (*F* = 28.22; *df* = 2, 57; *P* < 0.0001) and a greater number of eggs laid over 10 days (*F* = 23.61; *df* = 2, 57; *P* < 0.0001) (Fig. 3a,b). A similar pattern was observed from the group inoculated with *C. insecticola*. Finally, for both sexes, the inoculation with either *C. jiangsuensis* or *C. insecticola* yielded significantly larger body length, compared to the apo-symbiotic group (female: *F* = 39.46; *df* = 2, 57; *P* < 0.0001; male: *F* = 57.97; *df* = 2, 57; *P* < 0.0001; Fig. 3c,d).

## Discussion

The results of this study indicate that when *R. pedestris* were provided with field-collected soil, single bacterial species in family Burkholderiaceae was established in a majority of the insects. This pattern is consistent with the findings of previous studies. Kim et al. found that although soil in general contained three bacterial clades in Burkholderiaceae in South Korea, 45% of *R. pedestris* harbored single clade when reared on the field-collected soil^16^. *Riptortus pedestris* has a constricted region in the midgut, a narrow passage filled with a mucus-like matrix, which is known to sort specific bacteria^21^. This sorting organ serves as an important barrier to prevent the influx of non-symbiotic or pathogenic microbes during the environmental acquisition of symbiotic bacteria by *R. pedestris* from soil^21^. Indeed, soil typically contains highly diverse bacterial complex even at small spatial scales, for example, ca. 3.8 × 10^6^ species in one-gram soil^15^. In addition to the sorting mechanism, interspecific competitions between bacterial species are likely to affect which species would eventually colonize the midgut crypts of *R. pedestris*. A single bacterial species such as *C. insecticola* was dominantly established in the midgut of *R. pedestris* when the insects were co-inoculated with two bacterial species belonging to different genera in laboratory conditions^7^.

Along with the internal mechanisms described above, inevitably soil microbiota would serve as fundamental basis in determining the symbiotic bacterial community of *R. pedestris*. Therefore, it is essential to characterize soil microbiota and better understand ecological associations of bacterial community between soil and insect. However, this study provides limited information on the soil bacterial community because only diagnostic PCR analysis was conducted with soil samples. Nonetheless, our results confirm the presence of the target bacterial genera, all of which were detected from *R. pedestris*, in each soil sample analyzed. In addition to this basic validation, continuous research needs to include comparative analysis between soil and insect bacterial communities.

In this study, when insects were reared on field-collected soil, a total of 15 bacterial species in Burkholderiaceae were found in the midgut of *R. pedestris*. Therefore, the constricted region is selective toward symbiotic bacteria, but still allows the entry of diverse species in Burkholderiaceae to the midgut M4 region of *R. pedestris*. Among the bacteria detected, only four species, *C. insecticola*, *C. megalochromosomata*, *C. cordobensis*, and *C. udeis*, have been previously documented for their associations with *R. pedestris*^7^. In particular, this study reports for the first time that *C. jiangsuensis* was the most dominant species established in the midgut of *R. pedestris*. Moreover, the current research also evaluated symbiotic effects of *C. jiangsuensis* on the insects. This symbiotic bacteria significantly enhanced the development, growth, and reproductive potentials of *R. pedestris*, compared to the insect with no symbiotic bacteria. In addition to *Caballeronia* spp., recent findings also demonstrated the symbiotic effects by *Paraburkholderia fungorum* and *Pandoraea norimbergenesis* on *R. pedestris*^7^. Therefore, continuous effort is needed to investigate bacterial complex establishing symbiosis with *R. pedestris* and their associations the insects.

When interpreting the symbiotic bacterial diversity reported in this study, it is recommended to consider the following two aspects. First, culture-dependent methods were used to characterize the bacterial species diversity in *R. pedestris*. Thus, we cannot rule out the possibility that this approach might have underestimated the bacterial diversity because the culture conditions were not suitable for some bacteria species requiring more specific environments. In addition, even when the culture conditions of this study were optimal for most symbiotic bacteria, still taking three colonies per insect might have not been sufficient to represent the bacterial species diversity in *R. pedestris*. Second, the symbiotic bacteria were identified based on taxonomic assignment using the similarity-based search with the 16S rRNA genes. This methodology can entail the ambiguity of species identification including the lack of the gene sequence similarity with a matched type strain. Indeed, we did not include the data sets from 14 insects in the symbiotic bacterial diversity analysis due to the limitations with species identification. In addition, this approach cannot distinguish *C. insecticola* and *C. peredens* among the bacteria species detected. Nonetheless, it is also noteworthy that *C. insecticola* is well known for its symbiotic associations with *R. pedestris*, whereas no record exists for *C. peredens*. In future studies, culture-independent methods such as the NGS analysis need to be employed to obtain comprehensive and high-throughput sequencing data, thereby alleviating the limitations of the current study.

Interestingly, *C. jiangsuensis* is known to degrade an organophosphate insecticide, methyl parathion, in soil collected from Jiangsu Province, China^22^. Although methyl parathion is no longer available in many countries^23^, symbiosis with *C. jiangsuensis* may have important implications for pest management with regard to the development of insecticide resistance by *R. pedestris*. Kikuchi et al. demonstrated that symbiotic association with bacterial strains that degrade fenitrothion in soil could also confer insecticide resistance to *R. pedestris*^3^. Continuous applications of the synthetic insecticides could make insecticide-degrading bacteria more abundant in soil, which may in turn facilitate symbiosis with *R. pedestris*, conferring the insects with insecticide resistance^3^. Therefore, it is worthwhile to further evaluate whether symbiosis with *C. jiangsuensis* might confer insecticide resistance to *R. pedestris*, especially against organophosphates. In addition, the second dominant species was *C. megalochromosomata*, originally isolated from grassland soil in mountain, South Korea^24^. In Japan, *C. megalochromosomata* was demonstrated in a laboratory inoculation experiment to successfully establish in *R. pedestris*^7^; however, no study has been conducted to evaluate the biological effects of this bacterial species on the insect.

In summary, this study characterizes the species diversity of symbiotic bacteria establishing in *R. pedestris*, and the results indicate that this insect can establish symbiosis with at least a dozen of the bacterial species in family Burkholderiaceae. Given that soil bacteria communities are highly variable depending on soil properties^25^, flora^26^, and human activities such as cultivation^27^, it is important to continue investigating the symbiotic bacterial diversity by including a wider range of seasonal and geographical variations. Further studies are warranted to investigate the symbiotic effects of the unexplored bacterial species on *R. pedestris*, and reveal the evolutionary relationships between the two groups.

## Material and methods

### Soil samples

Soil samples were collected from the topsoil layer of forested areas near soybean fields in two regions in South Korea: Gwangju-si, Gyeonggi-do (37° 25′ 31.00″ N, 127° 19′ 40.00″ E) and Goesan-gun, Chungcheogbuk-do (36° 53′ 38.00″ N, 127° 49′ 21.00″ E). The two locations were confirmed with the occurrence of *R. pedestris* and the establishment of the symbiotic bacteria in the local populations^16^. In each region, a total of 10 soil samples (ca. 40 mL) were collected at least 5 m away from each other using disposable wooden chopsticks, and the samples were stored individually in 50 mL conical tubes. Soil samples were collected in mid-July 2021, during which the second-instar *R. pedestris* become abundant in South Korea^28,29^. This insect is known to acquire its symbiotic bacteria from soil mainly during the second-instar period^2,4^. Soil samples were brought to the laboratory, and stored at 4 °C^30^, before use in the experiments.

### Insect rearing

A laboratory colony of *R. pedestris* was originally established from wild individuals collected from wooded areas in Gyeonggi-do, South Korea (37° 27′ 4.11″ N, 127° 7′ 52.57″ E). From the insect colony, *R. pedestris* eggs were collected and incubated in clean breeding jars (100 mm × 40 mm (diameter × height)). The first-instar nymphs were transferred to new breeding jars with distilled water with 0.05% ascorbic acid (DWA). When they molted into the second instar, 10 individuals were randomly selected, and transferred to a clean breeding jar provided with field-collected soil (ca. 3 mL) and dried soybeans. Each soil sample was prepared separately and provided to the nymphs in the breeding jar. Soil was moisturized with DWA to facilitate the acquisition of symbiotic bacteria by *R. pedestris* nymphs from soil. Once *R. pedestris* developed into the third instar, they were transferred to clean breeding jars with DWA and soybeans. Insects were reared to adults under 26 ± 2 °C, 16L:8D, and 35–40% RH.

### PCR analysis for soil samples

Individual soil samples provided to *R. pedestris* were subject to DNA extraction using a DNeasy PowerSoil Pro kit (QIAGEN, Hilden, Germany) according to the manufacturer’s protocol. Then, diagnostic PCR was conducted using three primer sets to detect genus *Caballeronia* (SBE 160F and SBE 1400R)^16^, *Paraburkholderia* (Burk16SF and PBE822R)^16^, and *Burkholderia* (BCC370F and Burk16SR)^16^. The temperature profile for diagnostic PCR was as follows: 95 °C for 10 min, followed by 30 cycles of 95 °C for 30 s, 55 °C for 1 min, and 72 °C for 1 min^16^.

### Identification of symbiotic bacterial species

To identify bacterial species colonizing the midgut of *R. pedestris*, three adults per soil sample were randomly selected, yielding a total of 60 individuals. To minimize contamination, the external surface of the insects was sterilized with 70% ethanol before dissection^6^. Then, the sterilized insects were dissected with forceps in a petri dish filled with a phosphate-buffered saline (PBS). The midgut M4 region was carefully removed and rinsed with PBS, then homogenized in 100 μL of PBS. The homogenate was immediately spread on yeast extract–glucose (YG) agar plates^6^. After incubation at 26 °C for < 72 h, cultured colonies were grouped based on the similarity of their morphological traits. In general, there were up to two distinct morphological groups cultured from an insect. Within each group, colonies were randomly selected as needed, thereby including diverse morphological traits in the three colonies subject to further DNA analysis. DNA extraction from each colony was conducted using a MagListoTM 5 M Genomic DNA Extraction Kit (Bioneer Co. Ltd., Daejeon, South Korea) according to the manufacturer’s instructions. The DNA samples were analyzed for bacterial species identification as follows.

Nearly full-length (ca. 1400 bp) of the 16S rRNA gene from each bacterial colony was sequenced using universal primers 1492R and 27F (SolGent, South Korea)^31^. Based on EzBioCloud as 16S rRNA reference database, sequence similarity value was calculated by the pairwise sequence alignment algorithm for each colony, and the most closely-related type strain was recognized for taxonomic assignment^32,33^. When the similarity value was greater than 98.65%, the most closely related type strain was identified as the species of the given colony^33,34^. On the other hand, when individuals had colonies with the similarity levels equal to or below 98.65%, these individuals were not included in the species diversity analysis. In addition, irrespective of the similarity level, if multiple type strains yielded the same highest similarity in a colony, these individuals were also excluded from the data analysis. However, as an exception, *C. insecticola* and *C. peredens* were counted as ‘*C insecticola* or *C. peredens*’ because the 16S rRNA sequence of those two species is identical^35^. Then, phylogenetic tree was generated using MEGA 11 software with the identified bacterial species^36,37^. The phylogenetic tree was constructed using maximum-likelihood algorithm, and the topology of the tree was evaluated using bootstrap resampling method with 1000 replications^38,39^.

In addition, bacterial diversity was further analyzed by estimating pairwise sequence identity between 165 bacterial colonies isolated from *R. pedestris* using MEGA 11 software. The pairwise sequence identity was determined for all the bacterial colonies and clustered into groups by collapsing nodes with 0.01 of sequence difference. Each group was aligned and subjected to pairwise sequence identity estimation using maximum composite likelihood model. Then, the phylogenetic tree of the bacterial colonies was generated based on maximum likelihood algorithm.

### Symbiotic effects on *R. pedestris*

To evaluate the symbiotic effects of the most dominant bacterial species, *C. jiangsuensis*, this species was isolated, and used in the following experiments. In this test, three groups were included to evaluate the symbiotic effects of the most dominant species: (1) *R. pedestris* inoculated with *C. jiangsuensis* GJ1F1a [OQ152651], (2) inoculated with *C. insecticola* (RPE225), and (3) untreated. *Caballeronia insecticola* (RPE225) was included as a model organism that is known for its symbiotic effects on *R. pedestris*^8^. The bacteria were cultured at 30 °C overnight in YG media on a shaking incubator (180 rpm). Each cultured bacterial species was adjusted to 10^7^ cell/mL with DWA using spectrophotometer (GeneQuantum, Japan) and prepared on a cotton pad placed in a breeding jar. Then, ca. 50 s instar nymphs were transferred to the breeding jar for inoculation with the designated bacteria. After 24 h, the insects were transferred individually in clean breeding jars with soybean seeds and DWA. Insects were reared to adult under 26 ± 2 °C, 16L:8D, and 35–40% RH. During the nymphal period, the insects were checked every day for their developmental stages. A separate group of *R. pedestris* were reared to adults to record their body length and time duration to the first oviposition, and the number of eggs laid over 10 days. Insects were reared in the same laboratory conditions. For each group, 20 individuals were tested, and the data were compared among the three groups using ANOVA with Tukey’s HSD test (JMP, Version 12).

After the evaluation, all individuals were sterilized with 70% ethanol, and the M4 midgut section was dissected out to confirm the designated inoculation status. First, *C. insecticola* (RFE225) was confirmed with the detection of the green fluorescent protein (GFP) under fluorescence microscopy^8^. For *C. jiangsuensis*, the symbiosis was confirmed via two steps: diagnostic PCR with specific primers Burk16SF (5′TTTTGGACAATGGGGGCAAC3′) and Burk16SR (5′GCTCTTGCGTAGCAACTAAG 3′), and 16S rRNA sequencing with subsampled individuals. Finally, no inoculation was also validated through diagnostic PCR with Burk16SF and Burk16SR^5^. The temperature profile for the diagnostic PCR was the same as described above in the soil analysis. Based on this inoculation confirmation, one individual was not included in the developmental time data because it was found not being successfully inoculated with *C. jiangsuensis*.

### Supplementary Information

Supplementary Figures.

Supplementary Table S1.

Supplementary Tables.

## Data Availability

The 16S ribosomal RNA gene sequencing data reported in this study were deposited in GenBank under the accession numbers from OQ152642 to OQ152806 (Tables 1, 2; Supplementary Table S1).
