# Supplementary material for: Species diversity of environmentally-transmitted bacteria colonizing Riptortus pedestris (Hemiptera: Alydidae) and symbiotic effects of the most dominant bacteria
Source: Sci Rep. 2023 Sep 13;13:15166. doi: 10.1038/s41598-023-42419-0 (PMC10499786; doi:10.1038/s41598-023-42419-0)
Supplement: Supplementary file 1 — Supplementary Figures. [file 41598_2023_42419_MOESM1_ESM.docx]

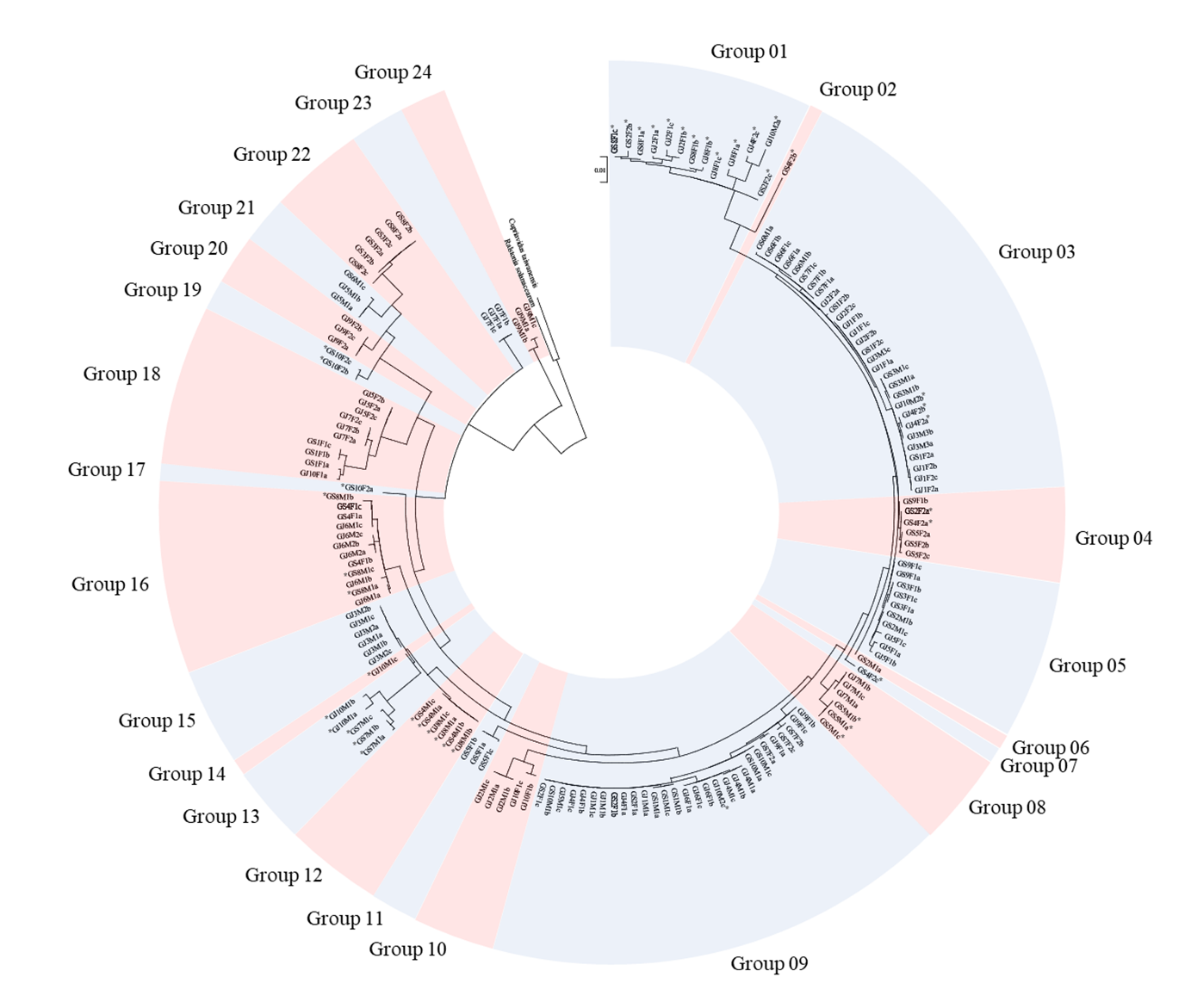


Supplementary Fig. 1. Phylogenetic diversity of obtained sequences from bacterial colonies isolated from midgut M4 of *Riptortus pedestris*. Phylogenetic tree was generated based on 16S rRNA sequences and each group was clustered by collapsing nodes with 0.01 of sequence difference in MEGA11. *Ralstonia* *solanacearum* and *Cuproavidus* *taiwanensis* were used as outgroup taxa. Asterisks indicate the bacterial colonies that were excluded in the species diversity analysis presented in Figure 1 and Tables 1 & 2.


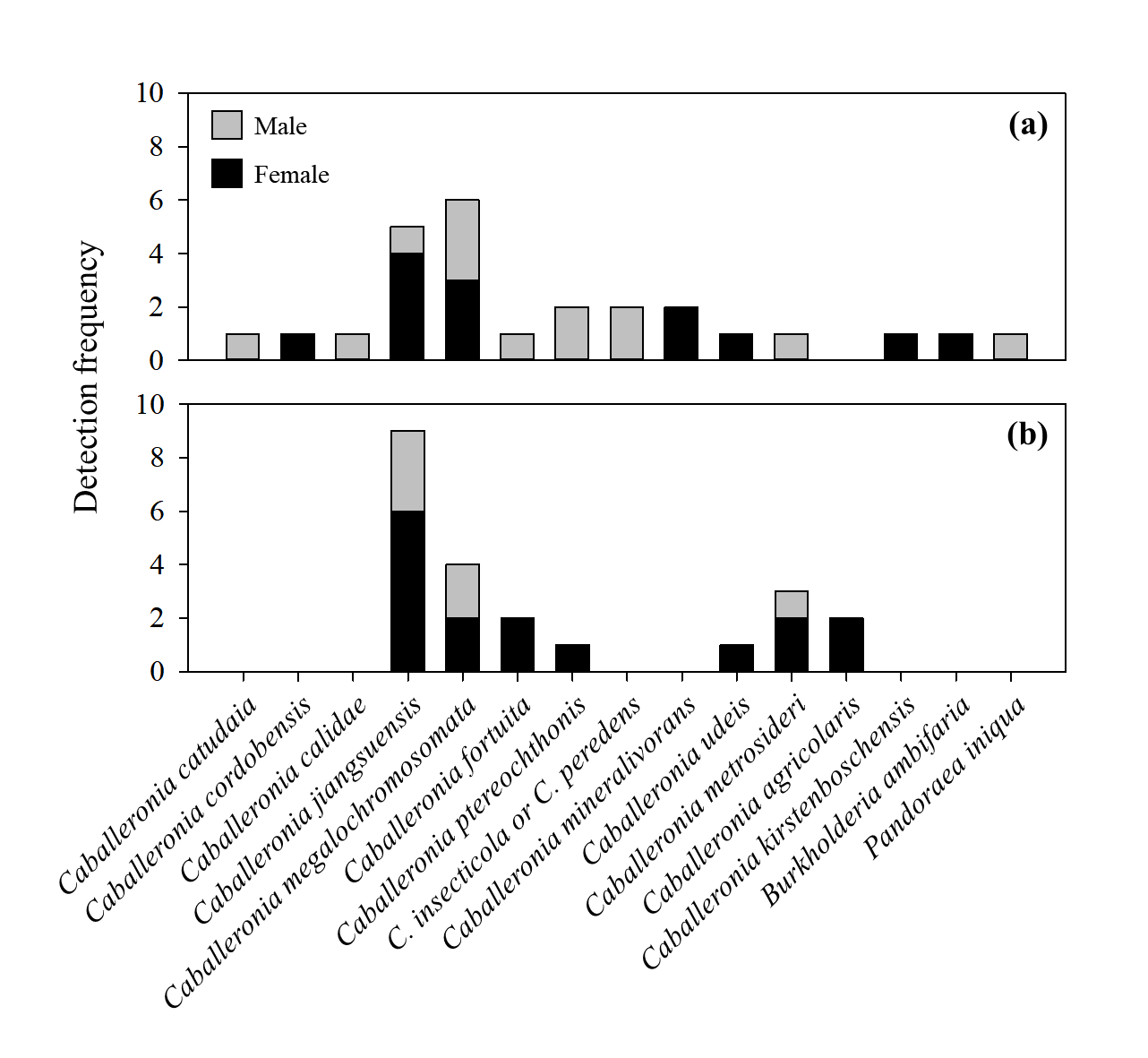


Supplementary Fig. 2. Detection frequencies of bacterial species isolated from the midgut of adult *Riptortus pedestris* in Gwangju (a) and Goesan (b). Note that detection frequency indicates the number of times being detected from insects and different colors indicate male or female of *R. pedestris*.
