# Supplementary material for: Species diversity of environmentally-transmitted bacteria colonizing Riptortus pedestris (Hemiptera: Alydidae) and symbiotic effects of the most dominant bacteria
Source: Sci Rep. 2023 Sep 13;13:15166. doi: 10.1038/s41598-023-42419-0 (PMC10499786; doi:10.1038/s41598-023-42419-0)
Supplement: Supplementary file 2 — Supplementary Table S1. [file 41598_2023_42419_MOESM2_ESM.docx]

Table S1. Symbiotic bacteria isolated from the midgut of *Riptortus pedestris* when insect had colony with the similarity level equal to or below 98.65 %^a^, or multiple type strains yielded the same highest similarity within colony^b^. These individuals were not included in the bacterial species diversity analysis.

| Insect  ID^*^ | Accession number | Type bacterial strain matched | Similarity with colony (%) | | |
| --- | --- | --- | --- | --- | --- |
|  |  |  | A | B | C |
| GJ2F1^a^ | OQ152660 − 152662 | *Caballeronia jiangsuensis* | 98.04 | 97.98 | 97.96 |
| GJ4F2^a^ | OQ152681 − 152683 | *Caballeronia jiangsuensis* | 99.29 | 99.21 | 96.91 |
| GJ8F1^a^ | OQ152714 − 152716 | *Caballeronia jiangsuensis* | 97.89 | 97.89 | 99.37 |
| GS2F2^a^ | OQ152747 − 152749 | *Caballeronia jiangsuensis* | 99.23 | 97.81 | 99.57 |
| GS8F1^a^ | OQ152795 − 152797 | *Caballeronia jiangsuensis* | 97.97 | 98.04 | 98.04 |
| GS4F2^a^ | OQ152765 − 152767 | *Caballeronia jiangsuensis* | 99.37 | 96.62 | 99.07 |
| GJ10M1^a^ | OQ152645 − 152647 | *C. insecticola* or *C. peredens*^†^ | 96.66 | 97.67 | 99.07 |
| GJ10M2^a^ | OQ152648 − 152650 | *Caballeronia jiangsuensis* | 97.88 | 99.50 | - |
|  |  | *Caballeronia megalochromosomata* | - | - | 99.43 |
| GS8M1^a^ | OQ152801 − 152803 | *Caballeronia ptereochthonis* | 98.93 | - | 98.79 |
|  |  | *Caballeronia fortuita* | - | 98.58 | - |
| GS10F2^a^ | OQ152729 − 152731 | *C. insecticola* or *C. peredens* | 98.58 | - | - |
|  |  | *Paraburkholderia madseniana* | - | 99.28 | 99.28 |
| GJ8M1^b^ | OQ152717 − 152719 | *Caballeronia megalochromosomata* | 99.00 | 99.00 | 99.00 |
|  |  | *Caballeronia pedi* | 99.00 | 99.00 | 99.00 |
|  |  | *Burkholderia novacaledonica* | 99.00 | 99.00 | 99.00 |
| GS4M1^b^ | OQ152768 − 152770 | *Caballeronia megalochromosomata* | 99.14 | 99.07 | 99.28 |
|  |  | *Caballeronia pedi* | 99.14 | 99.07 | 99.28 |
|  |  | *Burkholderia novacaledonica* | 99.14 | 99.07 | 99.28 |
| GS5M1^b^ | OQ152777 − 152779 | *Caballeronia grimmiae* | 99.36 | 99.22 | 99.22 |
|  |  | *Caballeronia calidae* | 99.36 | 99.22 | 99.22 |
|  |  | *Caballeronia pedi* | 99.36 | 99.22 | 99.22 |
| GS7M1^b^ | OQ152792 − 152794 | *Caballeronia fortuita* | 98.12 | 97.73 | 97.81 |
|  |  | *Caballeronia insecticola* | 97.97 | 97.73 | 97.81 |
|  |  | *Caballeronia peredens* | 97.97 | 97.73 | 97.81 |

^*^ GJ: Gwangju; GS: Goesan; F: female; M: male. ^†^ *C insecticola* and *C. peredens* are listed together because the16S rRNA sequence is known identical for the two species.
